# Supramaximal elevation in B-type natriuretic peptide and its N-terminal fragment levels in anephric patients with heart failure: a case series

**DOI:** 10.1186/1752-1947-6-351

**Published:** 2012-10-12

**Authors:** John YC Ting, Bruce A Pussell

**Affiliations:** 1 Anaesthesia Department, Wollongong Hospital, 348 Crown Street, Wollongong, NSW, 2500, Australia; 2 University of Wollongong, Wollongong, NSW, Australia; 3 Intensive Care Department, Coffs Harbour Health Campus, Coffs Harbour, NSW, 2450, Australia; 4 Nephrology Department, Prince of Wales Hospital, Barker Street, Randwick, Sydney, NSW, 2031, Australia

## Abstract

**Introduction:**

Little is known about the responses of natriuretic peptides to developing congestive heart failure in ‘anephric’ end-stage kidney disease.

**Case presentation:**

We present three consecutive cases of surgically-induced anephric patients in a critical care environment: a 28-year-old Caucasian woman (with congestive heart failure), a 42-year-old Caucasian woman (without congestive heart failure), and a 23-year-old Caucasian woman (without congestive heart failure). Our limited study data indicate that cut-off values advocated for B-type natriuretic peptide and its N-terminal fragment to ‘rule out’ congestive heart failure in two of our end-stage kidney disease patients (without congestive heart failure) are largely appropriate for anephric patients. However, our index (first) patient developed congestive heart failure accompanied by the phenomenon of massive and persistent elevation of these natriuretic levels.

**Conclusion:**

Our findings suggest that patients from the anephric subclass suffering from congestive heart failure will develop supramaximal elevation of B-type natriuretic peptide and its N-terminal fragment, implying the need for dramatically higher cut-off values with respective magnitudes of the order of 50-fold (B-type natriuretic peptide ~5780pmol/L; 20,000ng/L) to 100-fold (N-terminal fragment ~11,800pmol/L; 100,000ng/L) higher than current values used to ‘rule in’ congestive heart failure. Further research will be required to delineate those cut-off values. The role of our devised ‘Blood Volume – B-type natriuretic peptide feedback control system’ on ‘anatomical’ and ‘functional’ anephric patients led to significant mathematically-enriched arguments supporting our proposal that this model provides plausible explanations for the study findings, and the model lends support to the important hypothesis that these two groups of anephric patients inflicted with congestive heart failure should effectively have similar natriuretic response behavior.

## Introduction

Congestive heart failure (CHF) and renal failure (RF) act synergistically to increase the levels of B-type natriuretic peptide (BNP) and its co-secreted biologically inactive N-terminal fragment (NT-proBNP). These two cardiac neurohormones are mainly secreted from the ventricles and, to a lesser extent, the atria. They have an established role as useful diagnostic tests for CHF in both the pediatric and adult population, including RF patients [1-3].

RF and CHF represent two merging pathologies with a varying spectrum for speed of onset and severity. The intersection of cardiac and renal insufficiency is referred to as cardiorenal syndrome (Type 1 to 5) [4], which is CHF as a result of RF or vice versa. Cardiac dysfunction in end-stage kidney disease (ESKD) patients, whether acute or chronic, is often due to disorders of perfusion (ischemic heart disease) or to disorders of structure and function. The disorders of structure and function and CHF are often collectively termed ‘uremic cardiomyopathy’ which is commonly associated with left ventricle (LV) hypertrophy secondary to volume overload and hypertension (HT). Cardiac disease accounts for >50% of deaths in patients with ESKD [5].

The surgically-induced anephric (SIA) patients are a rarity. Despite this, health care providers such as general practitioners, emergency physicians, intensive care physicians and anesthetists will probably come across these anephric patients at some point in their working career. Little is known about the magnitude of natriuretic rise in response to developing CHF in this unique ‘anephric’ ESKD scenario.

We describe three consecutive SIA patients <60 years of age requiring high acute care in an Intensive Care Unit (ICU) or High Dependency Unit (HDU) at some point in their life between May 2004 and December 2009. The patients were all Caucasian adult women from a regional catchment population of 200,000 people in New South Wales, Australia. Using all information pertaining to their presentation obtained over varying periods of time (before and during their SIA states, and after having renal transplant in two of the three patients), including hospital records, results of diagnostic imaging and laboratory testing, we carefully studied each patient in both a prospective and retrospective manner specifically to determine the presence or absence of CHF.

The three subject patients were all on established hemodialysis (HD) thrice weekly (Monday-Wednesday-Friday schedule) for at least 30 days: the first session (Monday) was the dialysis session after a 72-hour inter-dialytic period whereas the second and third sessions were after a 48-hour period. All three patients were under the care of multidisciplinary teams; in particular under renal physicians to comprehensively manage their HD or post-renal transplant status.

The ‘zenith’ pre-dialysis, ‘nadir’ post-dialysis and ‘average’ inter-dialysis body fluid levels meant that their reflective blood samples were to be collected at (respectively) the start of a HD session, the end of a HD session and on a HD-free day. Whenever possible, hematological tests, biochemical tests, and specific hormone analysis for prolactin (ARCHITECT Prolactin assay; Abbott), BNP (ADVIA Centaur® BNP assay; Siemens Healthcare Diagnostics), and NT-proBNP (Elecsys® proBNP; Roche Diagnostics) were performed simultaneously on the same blood sample. Relevant data such as pre-dialysis ‘wet’ weight (kg) and post-dialysis ‘dry’ weight (kg) are used to estimate the net ultrafiltration volume removed as represented by a change in weight. Respectively, heart rate and respiratory rate are given in beats/minute and breaths/minute. Blood pressure (BP) in mmHg is recorded as systolic BP (SBP)/diastolic BP (DBP) with mean arterial pressure (MAP) computed from invasive arterial pressure waveform. Those same BP parameters were computed via the MAP = DBP + ⅓ (SBP – DBP) formula during electronic non-invasive BP measurements. ‘Severe HT’ is defined as SBP ≥180mmHg or DBP ≥120mmHg. ‘Resistant HT’ is defined as BP that remains above goal in spite of concurrent use of three anti-HT agents of different classes. The goal BP is less than 140/90mmHg in average risk HT patients.

Body mass index (BMI) is defined as (weight) ÷ (height)^2^ = kg/m^2^: normal (<25), overweight (25.0–29.9), and obese (≥30). Chronic kidney disease (CKD) is based on the abbreviated Modification of Diet in Renal Disease equation [6] to yield an estimated glomerular filtration rate (eGFR). We utilize the 2002 Kidney Disease Outcomes Quality Initiative of the National Kidney Foundation classification of the stages of chronic RF or CKD with increasing severity as CKD Stage 1 to 5. CHF can be LV systolic heart failure, LV diastolic heart failure, or both. In this paper, the term CHF is taken to be synonymous with ‘LV systolic heart failure’ with severity objectively classified by LV ejection fraction (LVEF) into normal, >50%; moderate, 35%–50%; and severe, <35%. LVEF values were obtained from all available echocardiograms performed on the patients.

## Case presentation

### Patient 1

A 28-year-old Caucasian woman (BMI 22, normal) with von Hippel–Lindau (VHL) syndrome had normal renal function before she abruptly became a permanent SIA patient (an ESKD state, CKD Stage 5) resulting in severe HT causing acute CHF (Event X) necessitating ICU admission (Day 0). Thus, her renal function just prior to the SIA state was normal. Her CHF became permanent and irreversible with the associated development of ‘intolerance to ESKD’ persisting for 5 years until her death (Event Y).

### Patient 2

A 42-year-old Caucasian woman (BMI 31, obese) with polycystic kidney disease (PKD) was admitted to HDU (Day 0) for post-operative care following subtotal parathyroidectomy for secondary hyperparathyroidism. She was already in ESKD state before she transitioned smoothly onto being a temporary SIA patient (also an ESKD state) 1.5 years earlier. Thus, her renal function just prior to the SIA state was ESKD. The ESKD component from her SIA state was temporary for a period of just over 2 years before her subsequent successful renal transplant (with resulting CKD Stage 3).

### Patient 3

A 23-year-old Caucasian woman (BMI 24, normal), the younger biological sister of Patient 1 and also inflicted with VHL syndrome, was admitted to HDU (Day 0) for post-operative care following a renal transplant. She abruptly went into ESKD after becoming a SIA patient. Thus, her renal function just prior to the SIA state was normal. She was in this temporary SIA state for 4.5 months until this successful renal transplant (with resulting CKD Stage 2).

**Figure 1:**
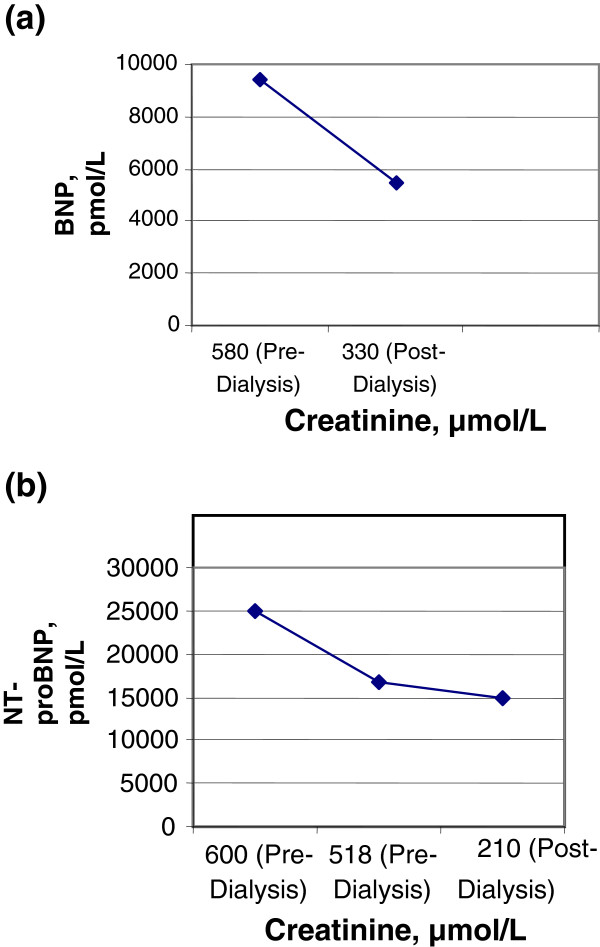
**a. B-type natriuretic peptide (BNP) data on Patient 1** (surgically-induced anephric [SIA], congestive heart failure [CHF]). **b. N-terminal fragment (NT-proBNP) data on Patient 1 **(surgically-induced anephric [SIA], congestive heart failure [CHF]).

For the interested reader, the clinical records on demographic and clinical characteristics of our subject patients are more fully detailed in their individual case reports (portrayed in Additional file 1: Appendix 1). The complete set of data collected in our study on echocardiograms and blood test results is depicted in Tables 1 and 2 respectively. In addition, BNP and NT-proBNP data from Patient 1 are depicted in Figure 1a and 1b respectively. All patients fulfilled the criteria for being in SIA state at some stage in their life. Our index (first) patient developed CHF. The remaining two patients did not develop CHF and they also had eventual renal transplants. This case mix of patients with and without CHF (on clinical grounds and objectively confirmed by echocardiograms) was necessary for analyzing cut-off values for natriuretic peptides to ‘rule in’ and ‘rule out’ CHF in anephric patients (respectively).

**Table 1:** Available echocardiogram results in chronological order

**Patient**	**CHF**	**LVEF% (Timing in relation to Day 0 presentation)**
1	Yes	(i) 15–20% (Event X / 0 day), (ii) 45–50% (+23 days), (iii) 30% (+6 months), (iv) 37% (+4.2 years), (v) 40% (+4.3 years), and (vi) 30% with a large mitral valve vegetation causing moderate to severe mitral regurgitation (Event Y / +5 years)
2	No	(i) 62% (+3 months)
3	No	(i) 60% (−3.4 years), (ii) 68% (−2.5 months), and (iii) 58% (+9 months)

CHF; Congestive heart failure, LVEF; Left ventricular ejection fraction.

**Table 2:** Available B-type natriuretic peptide, N-terminal fragment, and prolactin results in chronological order

**Patient 1 SIA stage**	**Creatinine, μmol/L**	**BNP, pmol/L**	**NT-proBNP, pmol/L**	**Prolactin, pmol/L**
**Blood samples (Status)**				
1 (Pre; +5 months)	630	N/A	N/A	27,561 [13,438]
2 (Pre; +5.5 months)	600	N/A	N/A	41,682 [20,323]
3 (Pre; +8.75 months)	580	9410 [32,563; >400]	N/A	37,398 [18,234]
4 (Post; +8.75 months)	330	5478 [18,958; >400]	N/A	37,923 [18,490]
5 (Pre; +10.75 months)	600	N/A	25,060 [212,370; >1200]	26,868 [13,100]
6 (Post; +10.75 months)	210	N/A	14,814 [125,540; >1200]	18,254 [8,900]
7 (Pre; +5 years)^β^	518	N/A	16,846 [142,765; >1200]	8087 [3,943]
^β^Blood sample obtained during terminal Event Y. Anti-hypertension methyldopa was ceased for one year prior to this.				
**Patient 2** SIA stage	Creatinine, μmol/L	BNP, pmol/L	NT-proBNP, pmol/L	Prolactin, pmol/L
Blood samples (Status)				
1 (Inter; +1 day)	763	79.2 [274; <225]	N/A	N/A
**Patient 2** Post-SIA stage	Creatinine, μmol/L	BNP, pmol/L	NT-proBNP, pmol/L	Prolactin, pmol/L
Blood samples (Status)				
1 (Post-Tx; +19 months)	136	52 [180; <201]	32.45 [275; <300]	1103 [538]
**Patient 3** SIA stage	Creatinine, μmol/L	BNP, pmol/L	NT-proBNP, pmol/L	Prolactin, pmol/L
Blood samples (Status)				
1 (Inter; -7 days)	588	14.8 [51.4; <225]	118.47 [1004; <300]	1389 [677]

(Status): (Pre = ‘zenith’ pre-dialysis, Inter = ‘average’ inter-dialysis, Post = ‘nadir’ post-dialysis, Post-Tx = (successful) post-renal transplant; Timing in relation to Day 0 presentation). N/A = nil available data.

For BNP and NT-proBNP, the two numerical values in parentheses refer to BNP or NT-proBNP values with unit ng/L; reference range to ‘rule in’ or ‘rule out’ CHF in unit ng/L as outlined in Table 4 in Discussion section of this article. The different reference range value (<201) quoted in Patient 2 Post-Tx is due to BNP cut-off values to ‘rule out’ CHF being stratified for GFR. For prolactin, the numerical values in parentheses refer to prolactin values with unit mIU/L with normal range <500 mIU/L.

SIA; Surgically-induced anephric, BNP; B-type natriuretic peptide, NT-proBNP; N-terminal fragment.

## Discussion

**Figure 2:**
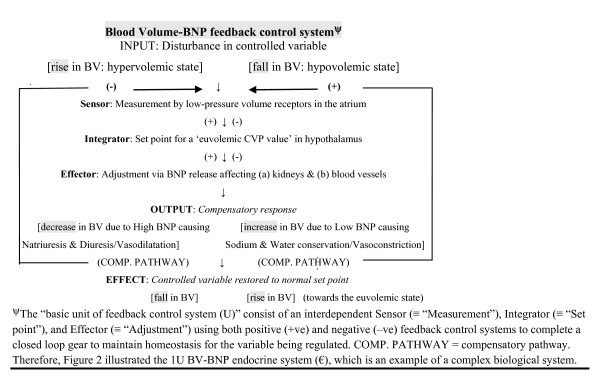
**Blood Volume – B-type natriuretic peptide feedback control system.** BV; Blood volume, CVP; Central venous pressure, BNP; B-type natriuretic peptide.

Predominantly based on concepts behind the feedback control systems, we devise the ‘Blood Volume (BV) – BNP feedback control system’ (Figure 2) with further discourse in Additional file 2: Appendix 2 to help provide plausible explanations for our findings.

For the interested reader, our intentional in-depth dissection on the study strengths, limitations and main findings (Additional file 3: Appendix 3) were tailored to provide some grounding for our recommended recruitment of minimum subject numbers for, and some aid to design, future study on a similar research topic. We also included an arbitrary evaluation on quantity and quality of the complete set of data collected in our study (Table 3) in Additional file 3: Appendix 3. Table 4 depicts currently available diagnostic cut-off values for BNP and NT-proBNP [2,7,8] to ‘rule in’ or ‘rule out’ CHF (stratified for age and GFR with data from patients with eGFR <15 mL/minute/1·73 m^2^ or those on renal dialysis therapy excluded).

**Table 3:** Quantity and quality of available B-type natriuretic peptide (BNP) and N-terminal fragment (NT-proBNP) results using arbitrary scales

**Pre-SIA stage**		**SIA stage**		**Post-SIA stage**	
					
Quant.	Qual.	Quant.	Qual.	Quant.	Qual.
-	NA	++	√√	NA	NA
-	NA	+	√	+	√√√
-	NA	+	√√√	–	NA

Quant. = Quantity of available results: – = Nil amount, + = Small amount, ++ = Moderate amount, +++ = Large amount

Qual. = Quality of available results (based on number of tests able to be done simultaneously on the same blood sample): √ = Fair, √√ = Good, √√√ = Excellent

SIA; Surgically-induced anephric, NA; Not applicable.

**Table 4:** **Cut-off values of B-type natriuretic peptide (BNP) and N-terminal fragment (NT-proBNP) as diagnostic tests to ‘rule out’ and ‘rule-in’ congestive heart failure (CHF)**^§^

**‘Rule out’ CHF**	**‘Rule in’ CHF**
BNP cut-off values to ‘rule out’ CHF: <100ng/L.	BNP cut-off values to ‘rule in’ CHF: >400ng/L.
When stratified for GFR,	
GFR >90mL/minute: <71ng/L.	
GFR >60–89mL/minute: <104ng/L.	
GFR >30–59mL/minute: <201ng/L.	
GFR <30mL/minute: <225ng/L.	
NT-proBNP cut-off value to ‘rule out’ CHF: <300ng/L.	NT-proBNP cut-off value to ‘rule in’ CHF.
	When stratified for age, When stratified for GFR,
	Age <50 year: >450ng/L. GFR >60mL/minute: >900ng/L.
	Age 50–75 year: >900ng/L. GFR <60mL/minute: >1200ng/L.
	Age >75 year: >1800ng/L.

^§^Reflects a nomogram version of Bayes’ Theorem, in diagnosis-uncertain patients (pre-test probabilities of ~50%), a likelihood ratio for a positive test result ≥10 is required to ‘rule in’ disease with reasonable certainty (post-test probability ≥90%) where a positive test result = (sensitivity) / (1 – specificity) = true positives / false positives ratio; whereas likelihood ratio for a negative test result ≤0·10 is required to ‘rule out’ disease with reasonable certainty (post-test probability ≤10%) where a negative test result = (1 – sensitivity) / (specificity) = false negatives / true negatives ratio).

CHF; Congestive heart failure, BNP; B-type natriuretic peptide, GFR; Glomerular filtration rate, NT-proBNP; N-terminal fragment.

From the study strengths, limitations and main findings expounded in Additional file 3: Appendix 3, the main message being alluded to is that (at least for this study) our results largely supported the quoted cut-off values advocated for BNP and NT-proBNP to ‘rule out’ CHF but not to ‘rule in’ CHF whereby dramatically higher cut-off values for BNP ~5780pmol/L (20,000ng/L) and NT-proBNP ~11,800pmol/L (100,000ng/L) will apparently be required. However, in contrast to our interpretation, using the recommended cut-off values of BNP <100ng/L and NT-proBNP <125ng/L for exclusion of CHF, Zuber *et al*. [9] have previously shown in 2009 that those chosen cut-off values fail to adequately ‘rule-out’ CHF in their 384 subject patients from a population with a high prevalence of CHF (50% with systolic heart failure and 8% with isolated diastolic heart failure). The BNP was a false negative in 25% of their patients and NT-proBNP was a false negative in 10% of their patients.

From Additional file 2: Appendix 2, we cater for better overall comprehension for general readers by alerting them to the following simplified explanatory remarks on the secondary endpoint of our study, which was to demonstrate supramaximal elevation of prolactin in Patient 1. This is based on analogous physiologic mechanisms that resulted in the demonstrated supramaximal natriuretic peptides levels (primary endpoint of our study) in the same patient. The supramaximal prolactin levels in this patient were due to the overall magnitude of rises in prolactin (arising from the effects of individual factors and/or causes of prolactin endocrine disorder that tend to increase prolactin production and/or decrease prolactin clearance) ‘far outstripping’ the overall magnitude of falls in prolactin (arising from the effects of individual factors and/or causes of prolactin endocrine disorder that tend to decrease prolactin production and/or increase prolactin clearance). To put our research in perspective, a crucial comment to make is that except to greatly aid in explaining the occurrence of supramaximal elevation phenomena for hormones in general, we acknowledge that the prolactin data per se in our manuscript do not help in pertaining to ‘rule in’ or ‘rule out’ CHF using biomarkers in any patients, not even in anephric patients.

In theory, the truly anephric state represents a unique position for research purposes because any ‘distorting interferences’ from a failing kidney are eliminated from consideration. These interferences refer mainly to the kidney (an integral component of the feedback control system) acting as: (1) an and/or the end target-organ for the relevant hormones; and/or (2) an and/or the organ contributing to metabolic clearance rate (MCR) for the relevant hormones. This ‘significant elimination’ holds true for the first interference (with the associated loss of the kidney compensatory pathway) but less so for the second interference because: (i) anephric patients are dependent on, usually, intermittent HD to keep them alive and thus providing them with an artificial means to intermittently and variably clear relevant hormones (namely, BNP and NT-proBNP); and (ii) there may be non-renal pathways (via other organs or tissues) variably contributing to MCR for these relevant hormones as well. This study should offer further research insights into the biological organization of the human body at the system level emphasizing adaptive and integrative mechanisms. This will advance our knowledge of physiology thus increasing our understanding of how our bodies function in health and disease.

For the interested reader, elucidation of utilizing a branch of mathematics known as combinatorics and step-by-step mathematical deduction applied to the BV – BNP feedback control system (as demonstrated in Additional file 2: Appendix 2) has led to plausible explanations for our study findings, and lent support to the important hypothesis that ‘anatomical’ and ‘functional’ anephric patients inflicted with CHF should effectively have similar natriuretic response behavior.

## Conclusion

Despite this study achieving the primary endpoint of demonstrating (sustained) supramaximal elevations of BNP and NT-proBNP in only one (obtainable) anephric patient inflicted with CHF (which suggested the need for dramatically higher BNP and NT-proBNP cut-off values for anephric patients in CHF with respective magnitudes of the order of 50-fold to 100-fold higher than the usual figures quoted to ‘rule in’ CHF), we were of the opinion that this should serve as a basis for future research aimed at further delineating the cut-off values for those hormones to diagnose CHF in anephric patients utilizing chemical biomarkers, thus enhancing the management of their cardiac disease. All these should broaden the knowledge of clinicians treating these patients and publicize the potential deleterious effect of not interpreting BNP and NT-proBNP tests appropriately in this setting. Based on elegant mathematical deductions, the BV – BNP feedback control system provides plausible explanations for our study findings, and lends support to the important hypothesis that ‘anatomical’ and ‘functional’ anephric patients inflicted with CHF should effectively have similar natriuretic response behavior.

## Consent

Written informed consents were obtained from the patients for publication of this case report. Copies of the written consents are available for review by the Editor-in-Chief of this journal.

## Competing interests

The authors declare that they have no competing interests.

## Authors’ contributions

JYCT designed the study, developed the analytic approach, worked on data collection, and wrote the first draft and subsequent writing of the paper. BAP contributed to the writing of the paper. All authors read and approved the final manuscript.

## Supplementary Material

Additional file 1 **Appendix 1.** Case reports. Click here for file

Additional file 2 **Appendix 2.** Blood Volume – BNP feedback control system. Click here for file

Additional file 3 **Appendix 3.** Study strengths, limitations and main findings [7,10-12]. Click here for file
