# Supplementary material for: Supramaximal elevation in B-type natriuretic peptide and its N-terminal fragment levels in anephric patients with heart failure: a case series
Source: J Med Case Rep. 2012 Oct 12;6:351. doi: 10.1186/1752-1947-6-351 (PMC3506524; doi:10.1186/1752-1947-6-351)
Supplement: Additional file 1 — Appendix 1. Case reports. [file 1752-1947-6-351-S1.pdf]

Appendix 1

Case reports

Patient 1

A 28-year-old woman with type 2 VHL syndrome (missense mutation P86S in exon 1 of the VHL gene) was admitted to ICU from the Emergency Department in May 2004 (Day 0) in acute pulmonary edema (APO) associated with severe type 1 respiratory failure (Event X). This was secondary to accelerated HT. The extreme ranges for the vital signs associated with Event X were BP from 170/110 to 180/110mmHg (MAP 130 – 133), heart rate (HR) from 130 to 170, and respiratory rate (RR) from 36 to 40. Her VHL syndrome was characterized by (and required various treatment modalities for): (1) visceral cysts in her pancreas and kidneys; and (2) the potential for malignant transformation in multiple organ systems; namely, renal cell carcinoma (RCC), retinal angiomas and spinal hemangioblastomas.

She required 6 days of supportive ICU care for Event X consisting of both invasive and non-invasive ventilation (NIV); continuous veno-venous hemodiafiltration (CVVHDF); and parenteral, oral, and topical anti-HT agents of different classes: glyceryl trinitrate, sodium nitroprusside (SNIP), hydralazine, phentolamine, phenoxybenzamine, metoprolol, captopril and amlodipine. She was subsequently discharged to the ward on a combination of multiple anti-HT agents (atenolol, irbesartan, lercadipine, minoxidil, clonidine, perindopril, spironolactone, prazosin, carvedilol and methyldopa) which were required to control her resistant HT resulting in typical readings BP ± 130/80mmHg (MAP ± 97).

Event X occurred 17 days after she became an SIA patient when intentional surgical removal of her remaining left kidney was carried out for recurrent RCC (right nephrectomy having been performed 4 years earlier for RCC). From Day 1 post-operative, she was commenced on an initial period of ‘gentle and frequent HD’ regimen (routinely advocated to prevent the dialysis disequilibrium syndrome complication attributed to cerebral edema). Her pre-operative baseline characteristics included: no clinical evidence of significant cardiorespiratory disease with BP 115/75mmHg (MAP 88), weight 69kg, height 1.75m, BMI 22, hemoglobin (Hb) 128g/L, creatinine 70μmol/L, and a previously normal echocardiogram with LVEF 60% (performed 2 years prior to Event X).

Based on a metaiodobenzylguanidine scan that was suspicious for a possible right adrenal pheochromocytoma, a right adrenalectomy was performed 5 weeks after Event X with histology showing ‘adrenal medullary hyperplasia of diffuse type’. This did not lead to any significant resolution of her resistant HT. Serial biochemical tests (which included plasma catecholamine studies) failed to detect any evidence for causes of secondary HT such as pheochromocytoma, hyperaldosteronism and hyperthyroidism. Serial imaging studies failed to detect any lesions causing secondary HT and any incidental lesions such as pituitary prolactinoma.

In fact, after her right adrenalectomy, she subsequently developed further episodes of HT emergencies: with BP elevated to 200/120mmHg (MAP 135). These ranged in severity from the milder ‘HT urgency’ type (6 months after Event X) to the more severe ‘accelerated HT’ of the

‘malignant HT’ type (HT encephalopathy with grand mal seizure; 3 months after Event X). The milder ‘HT urgency’ type required SNIP for severe HT and NIV therapy for mild APO; and the more severe ‘accelerated HT’ of the ‘malignant HT’ type required tracheal intubation for low Glasgow Coma score and phenytoin anticonvulsant therapy.

Her resistant HT was volume dependent and was observed by her renal physician to be partially responsive to instituting a relatively larger net ultrafiltration volume removal and progressively dropping the steady-state ideal ‘dry’ weight from ± 56kg 1 year after Event X to ± 53kg at 4 years (typical net ultrafiltration volume removal was 2.5 liter). This patient also had ‘intolerance to ESKD’. She developed amenorrhea, severe hyperprolactinemia (peak prolactin 41,682pmol/L; 20,323mIU/L), protein-energy malnutrition (with significant muscle wasting), intractable pruritus (needing ultraviolet B therapy), anorexia, profound weight loss, nausea and vomiting. The patient died at age 34 (Event Y) due to combined septic and cardiogenic shock. The infection stemmed from methicillin-resistant *Staphylococcus aureus* subacute endocarditis. She was given full supportive ICU care which included invasive ventilation, vasopressors and inotropes but rapidly succumbed to her critical illness within 48 hours.

In-patient and out-patient serial echocardiograms (reports chronologically listed below) obtained after she became a SIA patient confirm her to have established CHF with moderate to severe LV systolic HF. LVEF: (i) 15–20% (Event X / 0 day), (ii) 45–50% (+23 days), (iii) 30% (+6 months), (iv) 37% (+4.2 years), (v) 40% (+4.3 years), and (vi) 30% with a large mitral valve vegetation causing moderate to severe mitral regurgitation (Event Y / +5 years). The patient also had a coronary angiogram, 4-normal years after Event X, which shows normal coronary arteries and a measured ejection fraction of 40%. Natriuretic peptide measurements were carried out initially for diagnostic work-up with the first one performed around 9 months after Event X. Subsequent measurements were for both diagnostic work-up and research study purposes commencing after her study enrolment in January 2006 until Event Y in June 2009.

Patient 2

A 42-year-old woman with autosomal dominant PKD was briefly admitted to a HDU-type bed in March 2008 (Day 0) for post-operative care following uncomplicated elective subtotal parathyroidectomy for secondary hyperparathyroidism. Her pre-operative baseline characteristics include: no clinical evidence of significant cardiorespiratory disease, BP 131/64mmHg (MAP 86), HR 75, weight 88kg, height 1.69m, and BMI 31. She had her usual HD session for ESKD the day before surgery. Having enrolled this patient in our study just prior to this admission, we obtained her Day 1 post-operative ‘average’ inter-dialysis blood test results: Hb 108g/L, creatinine 763µmol/L, and BNP 274ng/L.

Her PKD was previously characterized by (and required various treatment modalities for) recurrent urinary tract infection, HT (previously treated with irbesartan and atenolol for 5 years but her HT resolved shortly after commencing HD), and progressive RF needing HD (commenced 2 years earlier). Bilateral nephrectomies were performed 1.5 years earlier for abdominal mass and/or discomfort due to the massively enlarged kidneys (thus becoming an SIA patient). She subsequently had a renal transplant 9 months later. Her PKD extrarenal manifestations included asymptomatic liver cysts but not intracranial aneurysms. Her other

significant medical history included hypothyroidism (on thyroxine replacement), and early menopause (onset 2 years earlier).

The patient had always tolerated HD sessions. She had a normal stress echocardiogram (with an LVEF of 62%) as part of the work-up 3 months before her successful renal transplant. She never developed CHF. Her condition was stable at 10.5 months post-transplant when she was followed up with a full panel of research blood tests: Hb 111g/L, creatinine 136μmol/L (eGFR 37mL/minute/1.73m2, CKD Stage 3), BNP 180ng/L, NT-proBNP 275ng/L, and Prolactin 538mIU/L.

Patient 3

A 23-year-old woman with VHL syndrome was briefly admitted to HDU in December 2009 (Day 0) for post-operative care following an uncomplicated renal transplant. She enrolled in our study in August 2009. Being the younger biological sister of Patient 1, she has a VHL syndrome genotype identical to that of her older sister. They manifested closely related phenotypic features for the disorder.

The patient has always been normotensive with pre-operative baseline characteristics: no clinical evidence of significant cardiorespiratory disease, BP 100/60mmHg (MAP 73), HR 60, weight 65kg, height 1.65m, and BMI 24. Her medical history was significant for right adrenalectomy for ganglioneuroma 14 years earlier (with subsequent right kidney atrophy); left partial nephrectomy 2 years earlier; left total nephrectomy 4.5 months earlier (thus becoming an SIA patient) with blood test just prior to this event showing Hb 130g/L and normal renal function with creatinine 67μmol/L. Her typical ideal 'dry' weight was 64.5kg with typical BP 115/70mmHg (MAP 85) and she usually tolerated her regular HD sessions well. Exactly 1 week before her renal transplant (SIA state), she was followed up with a full panel of research blood tests: Hb 119g/L, creatinine 588μmol/L, BNP 51.4ng/L, NT-proBNP 1004ng/L, and prolactin 677mIU/L. Her renal function 1 month after her successful renal transplant was creatinine 95μmol/L (eGFR 63mL/minute/1.73m2, CKD Stage 2). Due to difficult venipuncture, there was insufficient post-transplant blood drawn for hormonal analysis. Her echocardiograms confirm that she did not have CHF: LVEF (i) 60% (3 years before the SIA state), (ii) 68% (2 months after the SIA state), and (iii) 58% (9 months after her renal transplant).
