# Supplementary material for: Supramaximal elevation in B-type natriuretic peptide and its N-terminal fragment levels in anephric patients with heart failure: a case series
Source: J Med Case Rep. 2012 Oct 12;6:351. doi: 10.1186/1752-1947-6-351 (PMC3506524; doi:10.1186/1752-1947-6-351)
Supplement: Additional file 2 — Appendix 2. Blood Volume – BNP feedback control system. [file 1752-1947-6-351-S2.pdf]

Appendix 2

Blood Volume – BNP feedback control system

The following discussion is in reference to Figure 2 which depicted a typical 1 U Blood Volume – BNP feedback control system, whereby the symbol 'U' stands for the 'basic unit of feedback control system'. The control of a physiological 'state' or 'variable' such as BV is via a complex web utilizing adaptive and integrative mechanisms. The immediate control of the total body water (TBW) endocrine system (€TBW) is predominantly mediated by the 'thirst-antidiuretic hormone (ADH) mechanism'. Thirst affects the input of water and ADH affects the output of water. The delayed control of TBW (a minor control) is mediated by the endocrine system (€s) such as the renin-angiotensin-aldosterone system (RAAS, which also mediates 'control of BP' to some degree). The unit of this €TBW consists of: (i) sensors = osmoreceptors (monitor tonicity), low-pressure volume receptors (monitor BV), and high-pressure baroreceptors (monitor BP); (ii) integrator = hypothalamus; and (iii) effectors = thirst and ADH. Therefore the 'control of TBW' would also directly or indirectly lead to 'control of BP' (and vice versa) because both variables are controlled by RAAS to various degrees.

Similarly, the 'control of BV' variable (mediated by natriuretic peptides such as BNP in conjunction with RAAS) is affected to a varying degree by the 'control of TBW' and 'control of BP' variables. The '↑/↓ BV → ↑/↓ myocardial stretch or tension → ↑/↓ BNP release' homeostatic mechanism leads to the end-target organ effects of: (a) ↓/↑ BV (via (natriuresis and diuresis) / (sodium and water conservation) by kidneys) and (b) ↓/↑ peripheral vascular resistance (via (vasodilatation) / (vasoconstriction) on blood vessels). This signifies that our proposed homeostasis (with integrator = hypothalamus likely connected via autonomic neural pathway) acting through its compensatory pathways is invoked to help restore the disturbance in BV ('hypervolemic' / 'hypovolemic') to its 'euvolemic' set point resulting in improved diastolic relaxation (lusitropy) and decreased myocardial fibrosis. Measurements on BV status are largely carried out by the low-pressure volume receptors in the atrium (which essentially equates to central venous pressure clinical measurements). Likewise, detailed analysis for a 2 U hypothalamic–pituitary axis for prolactin hormone or a 3 U hypothalamic–pituitary–thyroid axis for thyroid hormones could also be carried out.

For a given €, the magnitude of its size and complexity would increase exponentially if a linear increase in the number of U were to occur due to the associated power-law increase in the number of 'controlled variables' along with their 'mini-components' (input, output, effect and compensatory pathway). The total number of possibilities arising from n mini-components when considering the '(↑ or +)' or '(↓ or –)' state (i.e. r = 2) for each mini-component is given by the permutations with repetition formula: $n^r = n^2$ from combinatorics. Let the symbol Σ denote 'the sum of'; and x*i* and y*i* denote 'n individual factors or causes of endocrine disorder' for *i* = 1, 2, 3,…, n that tend to have elevating or lowering properties, respectively, on the relevant hormone. The overall magnitudes of rises or falls in the particular hormonal output (O) (which is a controlled variable), such as BNP, NT-proBNP and prolactin hormonal concentrations, are governed by the net difference between the resultant effect from Σ (effects from xi that tend to increase O) and Σ (effects from yi that tend to decrease O). This overall resultant effect

stemming from the absolute difference between the n value for x*i* (nx) and the n value for y*i* (ny), namely |nx – ny|, that tends to increase or decrease O respectively would be some nonlinear function of this absolute difference of a synergistic nature. Then this overall resultant effect will be of ever greater cumulative rises or falls (of an exponential nature) in O when |nx – ny| is numerically >1 and constitutes an ever larger integer number.

The '↑/↓ BV causing ↑/↓ myocardial stretch or tension, resulting in ↑/↓ BNP release' is the main mechanism for BNP (and NT-proBNP) pulsatile co-secretion. Other mechanisms such as heart muscle cell damage from myocardial infarct will also lead to BNP and NT-proBNP release. There are two major cardiac and non-cardiac causes of BNP and NT-proBNP elevations as follows:

First, moderate increases in BNP (100–500ng/L) or NT-proBNP (250–1000ng/L): ventricular dysfunction, ischemic heart disease, pulmonary HT, acute pulmonary embolism, cor pulmonale, septic shock, renal insufficiency, liver cirrhosis, subarachnoid hemorrhage and hyperthyroidism.

Second, severe increases in BNP (>500ng/L) or NT-proBNP (>1000ng/L): decompensated heart failure (HF), pulmonary HT, acute pulmonary embolism and septic shock.

In addition to glomerular filtration, BNP is eliminated from plasma mainly through natriuretic peptide receptors and degraded by neutral endopeptidases. By contrast, it is possible that NT-proBNP is largely eliminated by glomerular filtration. Levels of both BNP and NT-proBNP are: elevated with ageing, higher in women than in men, higher in RF and CHF of greater severity, and higher in LV systolic HF than LV diastolic HF. Stage of HF (early versus late) and genetic polymorphisms may result in inter-individual variation of BNP and NT-proBNP. Obesity with and without CHF is associated with lower levels of both molecules; obesity with and without CHF is presumably attributed to non-hemodynamic factors such as BMI-related defect in natriuretic peptide secretion (from either ↓myocardial hormone release or ↓synthesis), and ↑BNP metabolism in adipose tissue either via peptide degradation or regulation of clearance receptors.

The end target-organs for BNP are the kidneys and blood vessels. These are associated with the 'kidney compensatory pathway' and 'blood vessel compensatory pathway' respectively. The SIA state corresponds to the loss of the kidney as (a) an end target-organ and (b) a compensatory pathway, although the contribution of the collective blood vessels as an end target-organ and compensatory pathway is still intact. The primary endpoint of our study was to demonstrate the supramaximal elevation of BNP and NT-proBNP in Patient 1. Computing from Figure 2 (together with the 'major cardiac and non-cardiac causes of BNP and NT-proBNP elevations'), this can be seen to be due to multiple x*i* (with no identifiable y*i*); namely: (i) CHF itself, (ii) decreased MCR for BNP and NT-proBNP, and (iii) total loss of kidney tissue acting as an end-target organ (but intact collective blood vessels acting as an end-target organ) with total disruption of 'kidney compensatory pathway' loop.

The secondary endpoint of our study was to demonstrate the supramaximal elevation of prolactin in Patient 1 as suggested by the persistently high prolactin values obtained between Event X (development of acute CHF) and Event Y (death of the patient). This was due to multiple x*i* (with no identifiable y*i*); namely, the systemic disorders of: (i) chronic RF, (ii) emotional stress,

(iii) epileptic seizures, (iv) pharmacologic factors (anti-HT dopamine synthesis inhibitors methyldopa), and (v) decreased MCR for prolactin. Both the mildly elevated prolactin levels in Patients 2 and 3 mainly reflect the decreased MCR of prolactin due to CKD Stage 3 and anephric status (needing intermittent HD) for each respective patient.

The nx = 3 in Patient 1 for Σ (effects from xi that tend to increase the 'outputs' of BNP and NT-proBNP) with resultant massive and persistent elevation of these two natriuretic peptides. Applying the nx = 3 minus ny = 0 calculation giving a 'relatively large' |nx – ny| value of 3 predicts the overall magnitude of rises to be consistent with the supramaximal elevation of these hormones as seen in our study. Similar calculation of a 'relatively large' |nx – ny| value of 5 for prolactin x*i* and y*i* in Patient 1 also showed that they act in concert to greatly increase and maintain the high prolactin 'output' in a synergistic manner to explain its supramaximal elevation.

'Functional' anephric states should occur in adult ESKD patients when their *in-situ* remnant kidney tissues have totally lost all their functions or have atrophied completely. One could extrapolate that these patients should behave physiologically in a similar manner to SIA patients. A corollary to this argument would result in the hypothesis that when ESKD patients develop CHF with supramaximal elevations of BNP and NT-proBNP, they are likely to be functionally anephric. The full significances of this hypothesis in adults are yet to be fully realized. Supramaximal hormonal elevations when observed in neonates, infants and children will undoubtedly be due to mechanisms similar to that of their adult counterpart; and with the full impact of this hypothesis lying in uncharted territories. These are exciting areas for future medical research.

Let us mathematically analyze the following statement in a logical manner: The defined parameters nx, ny, and |nx – ny| are applicable to both 'anatomical' and 'functional' anephric patients. Because one can safely assume that all supporting criteria for the statement to hold true are present in both sets of patients, then this 'common denominator' statement per se can provide intuitive non-contradictory explanations for the supramaximal elevation phenomenon in all anephric patients inflicted with CHF. This 'common denominator' statement thus lends support to our proposed hypothesis that 'anatomical' and 'functional' anephric patients inflicted with CHF should have similar natriuretic response behavior.

Footnote on 'An infant in temporary anephric and congestive heart failure state manifesting supramaximal elevations of natriuretic peptides'

In October 2010, we encountered the case of a 5-month-old male baby (weight 7kg) with out-of-hospital cardiac arrest (due to commotio cordis) requiring 30 minutes of cardiopulmonary resuscitation before return of spontaneous circulation. He developed multiple organ dysfunction syndrome (MODS) requiring full ICU supportive care. The ICU supportive care included therapeutic hypothermia between 33°C and 34°C for the first 48 hours, full invasive ventilation for 12 days for acute CHF with fractional shortening (FS) 31% on echocardiogram (normal >30%) while on multiple inotropic and vasopressor agents, and CVVHDF for 5 days from Day 3 to 7 for (anuric) acute kidney injury with peak creatinine 93μmol/L (20–50) on Day 8: this probably equates to the baby being a temporary 'functional' anephric patient. Blood tests on Day

7 showed: Hb 88g/L, creatinine 50µmol/L, and supramaximal elevation of NT-proBNP at 173,392ng/L (20,460pmol/L). By Day 10, the patient had not required dialysis for 3 days with blood tests showing: Hb 84g/L, creatinine mildly elevated at 58µmol/L (with good urine output), and lesser magnitude of supramaximal elevation of NT-proBNP at 98,476ng/L (11,620pmol/L). He continued to steadily improve before being extubated onto continuous positive airway pressure on Day 12 with eventual discharge from the pediatric ICU on Day 27. Follow-up of the patient at 12 months post-cardiac arrest revealed remarkable recovery from his MODS with possibly very mild and subtle residual cognitive dysfunction from the hypoxic ischemic encephalopathy, normal renal function (creatinine 23µmol/L at 8 weeks post-arrest), and normal cardiac function (FS 42% on echocardiogram at 7 months post-arrest).
