# Supplementary material for: Supramaximal elevation in B-type natriuretic peptide and its N-terminal fragment levels in anephric patients with heart failure: a case series
Source: J Med Case Rep. 2012 Oct 12;6:351. doi: 10.1186/1752-1947-6-351 (PMC3506524; doi:10.1186/1752-1947-6-351)
Supplement: Additional file 3 — Appendix 3. Study strengths, limitations and main findings [7,10-12]. [file 1752-1947-6-351-S3.pdf]

Appendix 3

Study strengths, limitations and main findings

Although open to interpretations, we subjectively grade Patient 1 as providing a 'moderate amount' of 'good' quality data on the behavior of BNP and NT-proBNP in an SIA patient with CHF. Patient 2 best provides a 'small amount' of 'excellent' quality data on the same hormones in a post-SIA patient (successful renal transplant, without CHF). Patient 3 best provides a 'small amount' of 'excellent' quality data on the same hormones in a SIA patient without CHF.

Based on the Australian population of 22.3 million in 2010 [10], 2257 patients were newly commenced on treatment for ESKD, an incidence rate of 101 per million people per year. There were 18,971 people (849 per million) receiving renal replacement therapy at the end of that year. Of these, there were 8382 with functioning renal transplant and 10,590 were receiving dialysis for ESKD. The commonest etiologies of ESKD are diabetic nephropathy, glomerulonephritis and HT accounting for 71% of all adult cases. The pool of 'true' anephric patients as the cause of ESKD can only be derived with certainty from the SIA patients, although it is eminently plausible that ESKD patients (with the passage of time) will progress into the 'functional' anephric state when all their remnant and/or scarred kidney tissue has atrophied and lost its blood supply and cellular function. The SIA patients are uncommon: they constitute only a minority group (around 1–5%) from the remaining 3071 (29%) adult ESKD cases receiving dialysis treatment. We estimate the total number of SIA patients nationwide as only between 30 and 150 people. Thus we consider our achievement to obtain the three subject patients for this study to be not insignificant, although we would prefer to recruit (probably with extreme difficulty) at least five anephric patients with CHF and five anephric patients without CHF.

All CKD Stage 5 patients (which include SIA patients) would be expected to have their BNP and NT-proBNP levels variably ameliorated by HD. With the molecular weight of NT-proBNP (8.5kDa) > BNP (3.5kDa), one would intuitively expect the ability of HD to clear those molecules to be in the reverse order (BNP > NT-proBNP) but for each molecule to still retain its 'zenith' pre-dialysis > 'average' inter-dialysis > 'nadir' post-dialysis blood levels. Worsening CKD stage would cause the NT-proBNP / BNP molar ratio values to be >1. However, many other factors such as changes in cytokine and volume status from HD may also alter the extrarenal clearance of BNP (and NT-proBNP) during dialysis.

The notion that 'time is needed to establish a steady state of intravascular volume and to allow the heart to sense the new steady state' [11] meant that the 'nadir' post-dialysis blood levels of BNP and NT-proBNP when taken at the more appropriate time of an arbitrary (for example) 30 minutes (instead of immediately) post-dialysis will more accurately reflect the 'true' nadir levels. As the 'timing of post-dialysis blood samples collection' factor in our study is neither standardized nor accurately known, this factor will influence the accuracy of the true values for 'nadir' post-dialysis blood levels for BNP and NT-proBNP. In addition, the quality of a HD session will depend on factors such as blood flow rates, frequency and duration of the session, (low-flux versus high-flux) membrane type and surface areas, and dialysate fluid type. The 'nadir' post-dialysis blood levels for the same hormones will again be affected because this quality is not standardized.

Because most laboratories employ either BNP or NT-proBNP assay kits, there are logistic difficulties in getting both tests done simultaneously on the same blood sample (preferred by our study). In Patient 1 (ESKD, SIA with CHF), separate day sample analysis revealed an almost identical reduction of BNP (41.8%) and NT-proBNP (40.9%) by HD. Paired separate day sample analysis also coincidentally revealed an almost identical 'zenith' pre-dialysis and 'nadir' post-dialysis NT-proBNP / BNP molar ratio value of 2·66 and 2.70 respectively. For comparison, same sample blood analysis on Patient 2 (CKD Stage 3, post-renal transplant without CHF) revealed the NT-proBNP / BNP molar ratio to be 0.62; and same sample blood analysis ('average' inter-dialysis) on Patient 3 (ESKD, SIA without CHF) revealed this ratio to be 8. This (overly) high 'steady-state' ratio of 8, which mainly reflects relative HD clearances of BNP and NT-proBNP, is due to calculation based on only one blood sample collected from Patient 3 with a (probably) spurious result of NT-proBNP level being three times the cut-off value to 'rule out' CHF. However, the absolute BNP value of Patient 3 when analyzed together with the other BNP value from Patient 2 (while she was in the SIA state) seem to indicate that the quoted cut-off values to 'rule out' CHF for BNP 225ng/L (65pmol/L), and (possibly) for NT-proBNP 300ng/L (35.4pmol/L), are largely appropriate for SIA patients. BNP and NT-proBNP values are generally lower in obese patients [7]. The role played by this statement in Patient 2 (BMI 31, obese) is unclear.

We did not elaborate on analytical imprecision of assays used, such as NT-proBNP cross-reactivity with bioactive BNP and its interassay coefficient of variation for the analyses in our study. In the grand scheme of things, this 'data validation' is comparatively undesirable because any discrepancies (for instance, the spurious NT-proBNP level in Patient 3 being (only) three times the cut-off value to 'rule out' CHF) that may have arisen from these statistical laboratory issues will be many order of magnitudes smaller than the supramaximal elevations of hormones that had occurred in Patient 1. Data from the n = 17 HD patients depicted in the figure from the 2004 study by Wahl *et al.* [12] have tantalizingly suggested that three of their patients – presumptively without CHF as, notwithstanding possible missed diagnosis of CHF in their patients, this study has no reported CHF diagnosis – have NT-proBNP values either to be around or higher than 100,000ng/L. Crucially, these 'supra-elevated' levels of NT-proBNP may well indicate that these three particular patients were more 'functionally' anephric than the remaining 14 patients, thus indirectly supporting our study proposal on the need to dramatically increase the cut-off value for (at least) NT-proBNP to 'rule-in', or even to 'rule-out', CHF in anephric patients to at least match this value.

To 'rule in' CHF with GFR <60mL/minute, the quoted BNP cut-off value in Table 4 is 400ng/L (115.6pmol/L), and the NT-proBNP cut-off value is 1200ng/L (141.6pmol/L). Despite our novel research data being quite limited and derived from only one patient (Patient 1), the persistent and non-transitory nature of the supramaximal pre-dialysis > post-dialysis elevations of BNP and NT-proBNP values (clearly depicted in Figures 1a and b), obtained at completely different times, were numerically much higher than the quoted cut-off values when this patient was inflicted with CHF. We strongly argue that this would seem to indicate that to 'rule in' CHF the cut-off values should be much higher for BNP, in the vicinity of 5780pmol/L (20,000ng/L), and for NT-proBNP, in the vicinity of 11,800pmol/L (100,000ng/L); as exemplified by the obtained BNP and NT-proBNP values in Patient 1 to either be extremely close to, or be above, those higher advocated cut-off values of ours. In addition, the non-occurrence of this natriuretic supramaximal

elevation phenomenon in Patient 3 (biological sister of Patient 1 similarly inflicted with identical VHL syndrome but without CHF) will help strengthen this argument by, for instance, almost totally excluding VHL syndrome giving rise to a theoretical rare (albeit never seen before) neuroendocrine ‘ectopic’ non-cardiac organ source(s) for large natriuretic peptide secretion.

We speculate here that the decrease in the values of BNP and NT-proBNP after a HD session in Patient 1 could also represent (transiently) improving CHF and not just solely be due to clearance of the hormones and/or reduction in plasma volume by HD. Both BNP and NT-proBNP are clearly increased in plasma from HD patients, with much higher concentrations for NT-proBNP, causing a mean (± standard error) NT-proBNP / BNP molar ratio of 28.0 (± 4.4) in the study by Wahl *et al*. [12]. After HD, this ratio increased to a mean value of 36.0 (± 6.8). In the case of HD (especially with low-flux membranes), Wahl *et al*. [12] observed an increase in plasma NT-proBNP despite experimentally showing elimination of NT-proBNP by HD by analyzing the dialysis fluid. They advocated that these varying behaviors could well be explained by the different sizes of BNP and NT-proBNP, by their different half-lives (20 minutes and 60–120 minutes, respectively), and (surprisingly and contrary to our belief) by both hormones seemingly being released into the circulation during HD sessions.
